# Soil N_2_O emissions with different reduced tillage methods during the establishment of *Miscanthus* in temperate grassland

**DOI:** 10.1111/gcbb.12570

**Published:** 2018-10-24

**Authors:** Amanda J. Holder, Jon P. McCalmont, Rebecca Rowe, Niall P. McNamara, Dafydd Elias, Iain S. Donnison

**Affiliations:** ^1^ Institute of Biological, Environmental and Rural Sciences (IBERS) Aberystwyth University Aberystwyth, Wales UK; ^2^ College of Life and Environmental Sciences University of Exeter Exeter UK; ^3^ Centre for Ecology & Hydrology Lancaster Environment Centre Bailrigg, Lancaster UK

**Keywords:** bioenergy, land use change, maize film, minimum till, *Miscanthus*, nitrous oxide, no till, pasture

## Abstract

An increase in renewable energy and the planting of perennial bioenergy crops is expected in order to meet global greenhouse gas (GHG) targets. Nitrous oxide (N_2_O) is a potent greenhouse gas, and this paper addresses a knowledge gap concerning soil N_2_O emissions over the possible “hot spot” of land use conversion from established pasture to the biofuel crop *Miscanthus*. The work aims to quantify the impacts of this land use change on N_2_O fluxes using three different cultivation methods. Three replicates of four treatments were established: *Miscanthus x giganteus* (Mxg) planted without tillage; Mxg planted with light tillage; a novel seed‐based *Miscanthus* hybrid planted with light tillage under bio‐degradable mulch film; and a control of uncultivated established grass pasture with sheep grazing. Soil N_2_O fluxes were recorded every 2 weeks using static chambers starting from preconversion in April 2016 and continuing until the end of October 2017. Monthly soil samples were also taken and analysed for nitrate and ammonium. There was no significant difference in N_2_O emissions between the different cultivation methods. However, in comparison with the uncultivated pasture, N_2_O emissions from the cultivated *Miscanthus* plots were 550%–819% higher in the first year (April to December 2016) and 469%–485% higher in the second year (January to October 2017). When added to an estimated carbon cost for production over a 10 year crop lifetime (including crop management, harvest, and transportation), the measured N_2_O conversion cost of 4.13 Mg CO_2_‐eq./ha represents a 44% increase in emission compared to the base case. This paper clearly shows the need to incorporate N_2_O fluxes during *Miscanthus* establishment into assessments of GHG balances and life cycle analysis and provides vital knowledge needed for this process. This work therefore also helps to support policy decisions regarding the costs and benefits of land use change to *Miscanthus*.

## 1 INTRODUCTION

Nitrous oxide (N_2_O) is a potent atmospheric greenhouse gas (GHG), and agriculture is the largest contributor of N_2_O to the atmosphere (IPCC, 2014; Reay et al., 2012; Smith et al., 2008). Soil management and tillage can impact on N_2_O emissions via the addition of fertilizers, plant residues, and changes to soil structure. These interventions influence microbial activity and thereby N_2_O emission through changes in water‐holding capacity, pore spaces, nutrient availability, and temperature (Butterbach‐Bahl, Baggs, Dannenmann, Kiese, & Zechmeister‐Boltenstern, 2013; Dobbie & Smith, 2003; Maag & Vinther, 1996; Smith et al., 2003). Similar to conventional crops, the establishment practices for perennial bioenergy crops such as *Miscanthus* and short rotation coppice also require weed control (normally via herbicide applications) and soil tillage during the cultivation. With little further soil disturbance or inputs during the crop's lifetime, this is a likely “hot spot” for GHG emission. The planting of perennial bioenergy crops is expected to increase in order to meet global greenhouse gas emission targets (Energy Technologies Institute, 2015; IPCC, 2007), and therefore, it is important that the establishment‐associated GHG impacts of land use change are understood.

To avoid competition with food, the use of economically marginal agricultural land (low grade and unprofitable) is preferred for biofuel crops (Lovett et al., 2009; Milner et al., 2016; Rathmann, Szklo, & Schaeffer, 2010). Agricultural grasslands make up a third of the utilized agricultural area across Europe, with higher proportions in some member states (e.g., Ireland, UK, Slovenia and Luxemburg) and could represent a key land use for conversion (Eurostat, 2018a). With changes in the management of grazing animals (Taube, Gierus, Hermann, Loges, & Schönbach, 2014; Xue, Lewandowski, & Kalinina, 2017), reduced profitability of grassland agriculture (DEFRA, 2017; Eurostat, 2018b), and uncertainly around agricultural policy reforms due to changes in the Common Agricultural Policy (European Commission, 2017), there is likely to be an increased interest in options for the diversification of grassland and especially more marginal grassland (Donnison & Fraser, 2016). The use of these lands for the growth of bioenergy crops including *Miscanthus* may be one option for this diversification and could also play a role in reducing overall agricultural GHG emission.

The *Miscanthus* genus (Greef & Deuter, 1993) is a perennial grass biomass feedstock with the commercially available sterile clone *Miscanthus x giganteus* (Mxg) attractive for its rapid biomass production, low nitrogen input requirements and ability to be grown on poorer agricultural soils (Clifton‐Brown, Schwarz, & Hastings, 2015; Lewandowski, Clifton‐Brown, Scurlock, & Huisman, 2000). Mxg is thought to be a natural hybrid of *Miscanthus sacchariflorus* and *Miscanthus sinensis* (Lewandowski et al., 2000) and newer *Sacchariflorus x Sinensis* hybrids are also being developed for growth on marginal sites (Lewandowski et al., 2016; Nunn et al., 2017). However, the impact on soil N_2_O emissions during the time of conversion from grasslands to *Miscanthus* production is generally poorly studied and requires attention to quantify the environmental sustainability of this crop.

Experiments to date on GHGs resulting from conversion to *Miscanthus* are centred around establishment into arable land rather than grassland, finding lower or similar levels of N_2_O emissions compared to annual crops (Davis, David, Voigt, & Mitchell, 2015; Oates et al., 2016; Smith et al., 2013). In contrast, there are no published studies documenting N_2_O emissions over the actual conversion process to *Miscanthus* from a grazed grassland, revealing a significant knowledge gap (Harris, Spake, & Taylor, 2015; Whitaker et al., 2018). Two studies have looked at N_2_O emissions from *Miscanthus* established on grass but have only measured fluxes in crops during their second and third growing seasons, and hence, uncertainties remain about the flux levels that can be expected. Saha et al. (2017) measured N_2_O in the second growing season for *Miscanthus* planted into grassland in various locations within a conservation area (in the USA) and found that N_2_O fluxes were six times higher for *Miscanthus* compared to the grassland in some places, but similar to the existing grassland in others. Roth, Jones, Burke, and Williams (2013) measured N_2_O fluxes in 2 and 14 year old *Miscanthus* (in Ireland) and compared this to established grassland (with a bi‐annual cut) finding that although the fluxes were higher in the 2 year old *Miscanthus*, this was not significantly different to the grassland site. In a review of the research to date on land use change to bioenergy crops, Whitaker et al. (2018) also highlight the need for more work relating to grassland transitions to *Miscanthus* and planting methods that may reduce emissions.

Establishment options exist for *Miscanthus*, and these could play a role in reducing the cultivation‐associated N_2_O emissions. Reduced tillage methods involving either planting directly into the soil without any form of ploughing (no till) or minimum tillage (cultivation to a shallow depth generally not more than 10 cm) are generally recognized to have the benefits of reduced soil erosion and water runoff and can lead to increases in soil organic matter and soil biological activity (Holland, 2004; Lal, Reicosky, & Hanson, 2007). However, the impact of no till cultivation on N_2_O emissions can vary and seems to be linked to soil type and water content (Chatskikh & Olesen, 2007; Grave et al.., 2018; Rochette, 2008). The use of a bio‐degradable film mulch has shown improved agricultural and economic performance of *Miscanthus* by increasing shoot density during establishment in cool temperate climates through increased soil temperature and conservation of moisture (Ashman, Awty‐Carroll, Mos, & Robson, 2018; Olave et al., 2017). Although not currently routinely employed with *Miscanthus*, the use of this type of film may expand as an aid in establishment, with increasing use of lower agricultural grade land at higher altitudes (Alexander et al., 2014; Clifton‐Brown et al., 2011; Lovett et al., 2009). Rapid commercial scaling of *Miscanthus* is also currently limited by the need for clonal propagation by rhizome so new interspecies seed‐based hybrids are being developed to maximize production of the crop (Clifton‐Brown et al., 2017; Lewandowski et al., 2016). These new varieties are now at the stage of large, pre‐commercial trials across Europe in marginal soils and have been developed in tandem with these new mulch film‐based agronomies (GRACE, n.d.).

In this work, we address both the N_2_O impacts of *Miscanthus* establishment on marginal upland semi‐improved grassland and the potential for different establishment methods to mitigate these. We compare the soil N_2_O emission during the cultivation and first two growing seasons of *Miscanthus x giganteus* and a novel *Miscanthus* hybrid planted using three different low soil disturbance methods. The trial site was at a higher altitude than generally used commercially for growing *Miscanthus* and was chosen as being representative of the kind of poorer quality semi‐improved grassland that is likely to be most in need of diversification opportunities under growing economic pressures.

The *Miscanthus* hybrid chosen (OPM‐10) was selected from a range of new seed‐based hybrids previously tested in multilocation trials across Europe (Lewandowski et al., 2016; Nunn et al., 2017). This particular hybrid (*Sacchariflorus x Sinensis*) has previously shown strong resilience in cooler environmental conditions and was thought likely to be suitable for these upland sites. This study has two main aims: firstly to compare soil N_2_O emissions between an established grazed pasture and the initial cultivation, planting, and first two growing seasons of *Miscanthus* and secondly to assess impacts on, and potential drivers of soil N_2_O emissions in different reduced tillage methods (no till, minimum till, and minimum till with the addition of a film mulch layer). To check establishment with the different cultivation methods, overwinter survivorship is also considered.

## 2 MATERIALS AND METHODS

### 2.1 Location and experimental plots

The experimental site is located near Cwmystwyth, Wales, UK (52.349°N 3.806°W), and is approximately 250 m a.s.l. on a 1:10 east facing slope. Formed over bedrock of interbedded mudstone/sandstone the soil texture is a clay loam/silty clay loam with a pH of 5. Field capacity was calculated to be approximately 38% volumetric water content following the methodology in Saxton and Rawls (2006). The land has been used for cattle/sheep grazing and silage crops for at least the last 25 years. No fertilizer or lime has been used since 2006 and since then the field has been used for extensive sheep grazing. An in‐field weather station (MiniMet Automatic, Skye Instruments Ltd, UK, and Delta‐T RG‐2 rain gauge, UK) recorded a total rainfall of 1,049 mm from June to December 2016 and 2,158 mm for January to December 2017. The precipitation recorded was within the normal range for upland grassland in the UK (2,000–3,000 mm/year), but very wet compared to the UK national annual average of 1,154 mm (Met Office, n.d.). Air temperatures ranged from −4 to 30°C over the 2 year period. The average minimum and maximum air temperatures (2 and 19.3°C, respectively) were slightly cooler and warmer than the UK 30 year average of 5.3 and 12.4°C (Met Office, n.d.).

**Figure 1:**
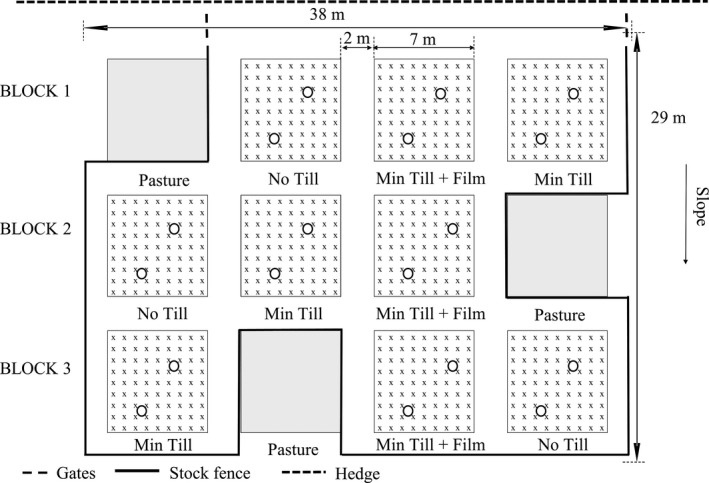
Plan of the experimental plot layout. “x” represents the planting positions and the circles represent locations of the static chamber collars. Each block contains a plot of existing undisturbed pasture (Pasture) and each of the three treatments: *Miscanthus x giganteus* rhizomes slot planted (No Till); *Miscanthus x giganteus* rhizomes planted with a minimum till method (Min Till); and *Miscanthus* hybrid OPM‐10 planted with a minimum till method and covered with a clear bio‐degradable film (Min Till + Film)

**Figure 2:**
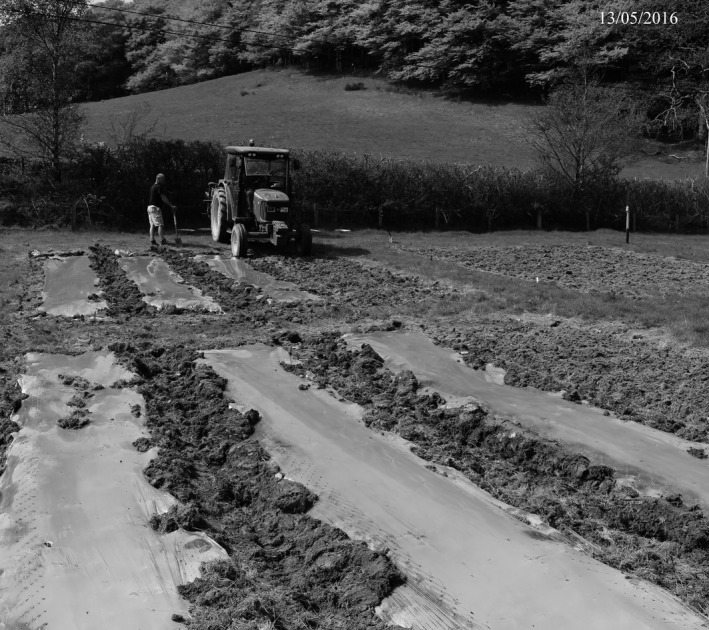
The bio‐degradable maize film layer being laid over the newly planted *Miscanthus* OPM‐10 hybrid plug plants on 13 May 2016

Twelve plots of 7 × 7 m were established in April 2016 using a randomized block design (Figure 1). The following treatments were included in each block: *Miscanthus x giganteus* (Mxg) planted via a no tillage method (No Till); Mxg planted via minimum tillage (Min Till); *Miscanthus* hybrid (OPM‐10) planted via minimum tillage and under a film layer (Min Till + Film); and an untreated plot of existing grass pasture (Pasture). Fencing allowed continuation of extensive sheep grazing on the pasture plots (Figure 1). Cultivation began on 14 April 2016 when all except the pasture plots were sprayed with glyphosate (1.5 kg/ha) to kill off existing vegetation. Planting of the *Miscanthus* plots took place on 13 May 2016 at a density of ~1.6 plants/m^2^ (81 plants per 49 m^−2^ plot). The No Till plots were slot planted with Mxg rhizomes by hand. The Min Till plots were rotavated to a depth of approximately 10 cm using a small tractor and rotavator. Three of the rotavated plots were planted by hand with Mxg rhizomes, and the other three were planted by hand with OPM‐10 as tissue cultured plug plants (plug size 4 × 4 × 8 cm). The OPM‐10 plants were then covered with a clear bio‐degradable maize film layer (Samco “Grey,” pinhole 20 aeration, Samco Agricultural Manufacturing Ltd, Limerick, Ireland; Figure 2). Each sheet of film covered two rows together which left one row in each plot uncovered. The film layer had mostly degraded after a month when the remainder was removed. In July 2016, any plants that had failed to establish were replaced with the appropriate Mxg rhizome or OPM‐10 plug.

### 2.2 Sampling of N_2_O soil emissions

Fortnightly static chamber gas sampling following the methods in Parkin and Venterea (2010) and Collier, Ruark, Oates, Jokela, and Dell (2014) began on 12 April 2016 and continued until 24 October 2017 (41 occasions). Two chamber collars, each covering an area of 0.12 m^2^, were inserted into the ground of each plot (Figure 1) to a depth of 5–6 cm. Collars were removed for cultivation, but otherwise remained in place throughout the study. In the Min Till + Film plots, holes to match the area of the collars were made in the mulch film to allow re‐insertion.

Equipment and sampling methodology followed were those in McCalmont et al. (2018). On each sampling occasion, chamber lids (area 0.0251 m^3^) with an external reflective surface and butyl rubber septum were attached to the collars with spring clamps. Every sampling event commenced with Block 1 between 10 and 11 a.m. (Alves et al., 2012); all treatments within the block were sampled at four time series before moving to the next block. Ten microliter gas samples from each chamber were taken at 0, 15, 30, and 45 min intervals and injected into preevacuated 3 ml glass vials sealed with rubber septa (Labco, Lampeter, UK). Concurrent chamber level air temperature was taken with a temperature probe (Testo 104, Testo Ltd. Hampshire, UK). Soil volumetric water content (ML3 soil moisture probe, Delta‐T Devices, Cambridge, UK, calibrated to the specific field soil) and soil temperature measurements (Testo 104, Testo Ltd, Hampshire, UK) were taken from within 1 m of each chamber using 10 cm hand‐held probes.

N_2_O concentrations were determined by gas chromatography (Perkin Elmer Autosystem XL Gas Chromatograph, USA), and the N_2_O fluxes were calculated using R version 3.2.3 (R Core Team, 2015) package “flux” v3.0–0 (Jurasinski, Koebsch, Guenther, & Beetz, 2014).

### 2.3 Soil sampling

Precultivation soil samples were taken in April 2016, followed by regular monthly soil samples (one from each plot) to a depth of 30 cm from June 2016 until October 2017. Samples were taken using a 4.8 cm internal diameter split tube soil auger (Eijkelkamp Agrisearch Equipment BV, Giesbeek, The Netherlands) and separated into 0–15 and 15–30 cm depths. Soil cores were subsampled for use in different analyses.

Fresh subsamples (5 g), used to assess nutrient availability, were mixed on a shaking table with 25 ml 1 M solution of KCl (potassium chloride) and then filtered (150 mm diameter hardened ashless filter papers, Whatman, UK) into 250 ml sterilin bottles and frozen at −20°C. The samples were later defrosted and analysed for nitrate (NO_3_^−^) and ammonium (NH_4_^+^) using continuous flow colorimetry with an AA3 HR AutoAnalyzer (SEAL Analytical Ltd, Southampton, UK).

Bulk densities for the two depths were calculated (following the method outlined in Emmett et al. (2008)) for the samples taken in April 2016, June 2016, March 2017, and July 2017 to account for changes occurring due to the tillage.

Gravimetric moisture was calculated from oven drying subsamples to constant weight (at 105°C) and then converted to volumetric water content using the bulk density measurement.

### 2.4 Global warming potential

To assess the impacts of cultivation driven N_2_O fluxes on previous estimates of the Global Warming Potential (GWP) costs per ha of biomass production (Hastings, Mos, & Yesufu, 2017), the total sum of the N_2_O fluxes for each of the cultivation methods was converted to CO_2_ equivalent (CO_2_‐eq) and put into the context of a simulated 10 year crop lifetime.

Daily N_2_O totals were created by multiplying the mean hourly fluxes (mg N_2_O m^−2^ hr^−1^) by 24 and converting to Mg/ha. Linear interpolation was used to fill in the gaps between the 41 fortnightly values, and results for each treatment were summed to a total flux following the method in McCalmont et al. (2018). Finally, totals were converted to CO_2_‐eq using IPCC (2007) conversion factor of 298.

Carbon intensity of producing biomass (including crop management, harvest, transport, and fuel preparation) over the lifetime of the crop was based on value of 4.40 g CO_2_‐eq/MJ (Hastings et al., 2017). This was converted to Mg CO_2_‐eq/ha using yield estimate of 12 Mg DM ha^−1^ year^−1^, or 120 Mg DM/ha for the full 10 year period (Larsen, Jørgensen, Kjeldsen, & Lærke, 2014). Whilst yields can vary and are typically reduced at the start and end of a crops’ lifetime, the figure used is taken as a representative mean for the 10 year time span, being at the lower end of a range of reported mean yields (Clifton‐Brown, Stampfl, & Jones, 2004; Larsen et al., 2014; McCalmont et al., 2018). The energy content used was 17.95 GJ/Mg DM (Felten, Fröba, Fries, & Emmerling, 2013).

### 2.5 Statistical analysis

Data were analysed using R version 3.2.3 (R Core Team, 2015). Cumulative N_2_O fluxes and over winter plant survivorship were tested using ANOVA and Tukey HSD posthoc tests with tillage (cultivation method) as the fixed factor and the random effect of block. Baseline fluxes (recorded on 12 April 2016) were compared with fluxes on 11 April 2017 using ANOVA with sample date and tillage as the fixed factors, the random effect of block, and a cube transformation to improve model residuals. As the two growing seasons represented very different stages in the establishment, N_2_O fluxes for the two seasons were tested separately and statistics were carried out on cube transformed data. To explore potential drivers of N_2_O emissions, Akaike's information criterion (AIC) was used for selection of linear models with the random effects of block and sample date and fixed factors of: tillage; NO_3_^−^ and NH_4_^+^ (each to a depth of 15 cm); air temperature; soil temperature (0–10 cm depth); and water‐filled pore space, with fixed factor selection restricted to avoid cocorrelated factors (air and soil temperature) within a single model (R packages “nlme” (Pinheiro, Bates, DebRoy, & Sarkar, 2017) and “MuMIn” (Barton, 2018)). In addition, impacts of tillage method on these drivers were explored using ANOVA and Tukey HSD post hoc tests using tillage method as a fixed factor and the random effect of block and sample date.

## 3 RESULTS

### 3.1 Establishment

In the OPM‐10 plots, 15% of the hybrids planted under film failed to establish after the initial planting, whereas all of the hybrid plants (100%) that were outside of the film failed. Across all the Mxg plots, 33% of the No Till treatment and 29% of the Min Till treatment failed to establish. All gaps were replaced in July 2016, and the survivorship after the first winter was 87% for the hybrid plants, 83% for the No Till treatment, and 78% for the Min Till treatment. There was no significant difference in the survivorship between the different treatments (*F*_2,5_ = 3.96, *p* = 0.09).

### 3.2 N_2_O fluxes

**Figure 3:**
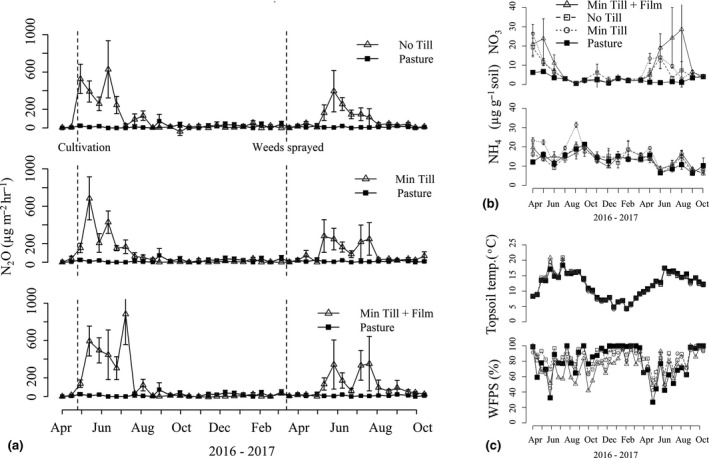
(a) Mean N_2_O flux over the sampling period (12 April 2016 to 24 October 2017) for the no tillage (No Till), minimum tillage (Min Till) and minimum tillage with film (Min Till + Film) treatment in comparison with the established pasture control (Pasture). The dotted lines show the time of cultivation in 2016 and the herbicide sprayed in 2017. (b) Mean levels of NO_3_^‐^ and NH_4_^+^ in soil samples (0–15 cm depth) taken monthly from June 2016 to October 2017. (c) The mean soil temperature (0–10 cm depth) and water‐filled pore space (WFPS; 0–15 cm depth) across the treatments. The error bars in all the charts show the standard error of the mean

**Figure 4:**
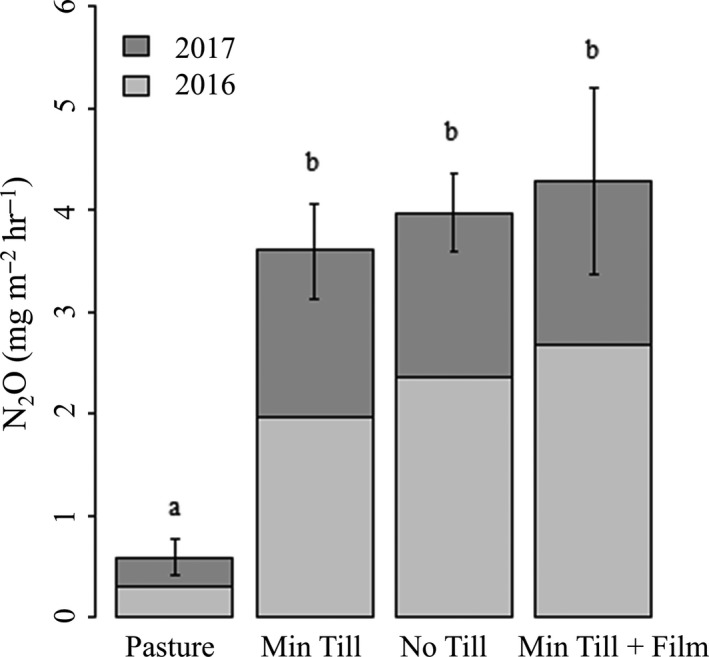
Mean cumulative N_2_O flux from 12 April 2016 to 24 October 2017. Error bars show standard error of the mean. The same letter indicates nonsignificant difference based on post hoc testing of the significant main effect of treatment

N_2_O spikes were observed in all the *Miscanthus* plots compared to the pasture control, regardless of tillage method (Figure 3a). However, whilst cumulative N_2_O fluxes over the 18 month period were higher under *Miscanthus* cultivation compared to the retained grassland controls (*F*_3,7_ = 8.51, *p* = 0.01) posthoc testing confirmed no significant difference in fluxes between the tillage methods for the *Miscanthus* (Figure 4).

Gas samples taken on 11 April 2017 from all the treatments and control showed a higher N_2_O flux (*F*_1,14_ = 13.83 *p* = 0.00) than was recorded in the preconversion baseline samples taken on 12 April 2016. However, fluxes were low in both instances (Figure 3a). On 11 April 2016, there was a zero flux rate in all the designated treatments (preconversion) with the exception of the No Till treatment, where a mean flux of 0.01 mg N_2_O m^−2^ hr^−1^ was recorded. A year later (12 April 2017), mean fluxes ranged from 0.01 to 0.03 mg N_2_O m^−2^ hr^−1^. There was no significant difference between the treatments at either of these time points.

The highest N_2_O peak recorded over the period (882.9 μg m^−2^ hr^−1^ on 19 July 2016) was seen in the Min Till + Film plots in the first growing season. The highest peak flux rates (μg N_2_O m^−2^ hr^−1^) recorded for the other treatments were as follows: Min Till 684.22 on 24 May 2016; No Till 628.10 on 22 June 2016; and Pasture 73.13 on 9 September 2016, again all in the first growing season. Flux rates reduced over the winter months but increased again in the tillage plots during the second growing season (Figure 3).

### 3.3 N_2_O drivers

Temperature and nutrient levels varied with season and between plots (Figure 2b,c). Model selection was conducted on each growing season separately but the same combination of drivers in each season achieved the closest fit with *R* ^2^ (marginal) values of 0.40 for the 2016 season and 0.39 for the 2017 season. The best combination of fixed factors for both growing seasons suggests that N_2_O fluxes were positively driven by NO_3_^−^ (0–15 cm depth) and soil temperature (0–10 cm depth), as well as tillage (Equation 1, asterisks denote interactions between the factors):

(1) $${\text{NO}}_{3}^{-}+\text{soil}\;\text{temperature}+\text{tillage}+{\text{NO}}_{3}^{-}*\text{soil temperature}+{\text{NO}}_{3}^{-}*\text{tillage}+1$$

Reflecting model selection, differences were found in soil temperature and NO_3_^−^ levels between the treatments in both growing seasons. The Min Till + Film and No Till treatments had higher soil temperatures than the Pasture (*F*_3,115_ = 3.97, *p* = 0.03) for the first growing season. However, for the second season, when differences are more apparent in plant morphology between *Miscanthus* and the Pasture grass, all the cultivated treatments had lower soil temperatures than the Pasture (*F*_3,125_ = 22.61, *p* < 0.001). Differences were also found in levels of NO_3_^−^ in treatments with extra disturbance and the addition of the film layer. In the first growing season, the Min Till + Film treatment had higher levels of NO_3_^−^ than the Pasture (*F*_3,115_ = 4.13, *p* < 0.001). This trend was also found in the second season where the Min Till + Film treatment had the highest levels compared to all the other treatments (including Pasture). In addition, the Min Till treatment was also higher than the Pasture (*F*_3,125_ = 10.54, *p* < 0.001). Although the potential driver of water‐filled pore space was not selected for in the best model combination, the Min Till + Film treatment was drier than all the other treatments during the first growing season (*F*_3,115_ = 12.02, *p* < 0.001). However, this was not observed in the second season where the Pasture was drier than the No Till and Min Till treatments, although the Min Till + Film treatment was drier than the No Till treatment (*F*_3,125_ = 9.39 *p* < 0.001). All the plots were above field capacity for the majority of the sampling period.

### 3.4 Global warming potential

The N_2_O emission resulting from cultivation (differences from grassland control) equated to a GWP (Mg CO_2_‐eq/ha) of 3.91 ± 0.13 for the No Till treatment, 3.57 ± 0.12 for the Min Till treatment and 4.90 ± 0.18 for the Min Till + Film treatment. The carbon cost of biomass production for *Miscanthus* over a 10 year crop lifetime was estimated as 9.49 Mg CO_2_‐eq/ha. When the mean N_2_O land use conversion cost of 4.13 Mg CO_2_‐eq/ha is added to the lifetime cost of production the overall cost of 13.62 Mg CO_2_‐eq/ha represents an increase of 44%.

## 4 DISCUSSION

This study highlights that regardless of cultivation method the establishment of *Miscanthus* on grassland is associated with increased N_2_O fluxes compared to uncultivated, unfertilized pasture. There are no other studies capturing fluxes during the initial cultivation for *Miscanthus* but studies of grassland tillage for reseeding do show similar flux levels to those we recorded in the *Miscanthus* cultivation (Drewer et al., 2017). This suggests that it may be the land disturbance itself and the residues of the previous crop rather than the following crop that is driving these increased emissions. The N_2_O fluxes from the retained pasture in this study were also similar to mean fluxes found across a number of European grazed and fertilized grasslands (Flechard et al., 2007).

N_2_O emissions have been considered to be a small part of the overall GHG balance in established plantations, with highest reported values (excluding cultivation/conversion) contributing 6% of total GHG balances (Dondini et al., 2016; Robertson et al., 2017). Our study shows that the land use conversion cost of N_2_O (4.13 Mg CO_2_‐eq/ha) represented approximately 30% of the total CO_2_‐eq. cost of producing the energy in the crop over 10 years (13.62 Mg CO_2_‐eq/ha). Whilst more studies are needed to understand potential impact over a wider range of sites (and soil types), this work does clearly show the importance of taking this initial increase in N_2_O into account when calculating GHG balances relating to land use change. However, it should be noted that this is a one‐off cost and the relative magnitude of its impact per unit of energy produced by the crop reduces in the long run. Yield predictions suggest that the life span of a commercial *Miscanthus* crop could be around 15 years depending on site conditions (Clifton‐Brown et al., 2015). Even including the carbon cost of the increased N_2_O fluxes during land conversion from grassland to *Miscanthus*, the GWP of the energy produced over a 10 year crop lifetime (6 g CO_2_‐eq/MJ) is lower than estimates for producing energy from coal (121 g CO_2_‐eq/MJ) and natural gas (59 g CO_2_‐eq/MJ; Hastings et al., 2017).

The initial soil N_2_O fluxes we recorded are in line with the prediction made by Roth et al. (2013) who suggested that fluxes may be higher during earlier stages of cultivation than from under 7 month old *Miscanthus*. However, whilst they found no significant difference between pasture N_2_O emissions and *Miscanthus*, our results did show a significant difference and higher peak flux rates. The deeper plough depth in Roth et al. (2013) may have had an impact, allowing more time for N_2_O to be reduced to N_2_ before reaching the surface (Baggs, Rees, Smith, & Vinten, 2000), but higher fluxes are just as likely to be the result of different edaphic and climatic site conditions. The water‐filled pore space in our study was in the optimum range for N_2_O emissions (~80%; Butterbach‐Bahl et al., 2013; Maag & Vinther, 1996), and rainfall at the site was above the UK national average which may in part have also contributed to the higher fluxes and highlights the need for more work across a range of climatic conditions.

Whilst N_2_O fluxes can vary between years (Drewer, Finch, Lloyd, Baggs, & Skiba, 2012; Jorgensen, Jorgensen, Nielsen, Maag, & Lind, 1997), the trend for a reduction in the second year after establishment seen in this experiment (despite higher second year early season fluxes) is in line with Roth et al. (2013) and fits with the generally low fluxes reported for mature *Miscanthus* plantations in studies by Gauder, Butterbach‐Bahl, Graeff‐Hönninger, Claupein, and Wiegel (2012) and Drewer et al. (2012). This suggests that fluxes in mature crops are likely to be lower than those recorded here for the conversion period, a point also noted in review by Whitaker et al. (2018).

Models predicted around 40% of the variance, values higher than those reported by Roth et al. (2013), where only 27% of fluxes could be explained by a large number of factors and Gauder et al. (2012) where soil temperature explained less than 10%. N_2_O fluxes are known to be volatile so higher frequency monitoring of N_2_O fluxes (in this study once every 2 weeks) with chambers, or continuous monitoring with the capture of larger ground areas via eddy covariance methods may reveal more about N_2_O drivers (Alves et al., 2012; Jones et al., 2011). The limited spatial nature of the chambers may be a reason that an effect of periods of grazing was not seen in the N_2_O emissions. However, the plots were extensively grazed and Flechard et al. (2007) found that whilst grazing tended to increase N_2_O emissions the effect was not clear and more noticeable only on fields that were also artificially fertilized.

Model selection showed NO_3_^‐^ and soil temperature as well as tillage to be significant in predicting fluxes. The use of herbicide to control weeds (used during initial cultivation and in mid‐March of the second year) provided plant material with the likely effect of stimulating microbial activity through increased carbon and substrate for nitrification/denitrification (Baggs et al., 2000; Huang, Zou, Zheng, Wang, & Xu, 2004; Palmer, Forrester, Rothstein, & Mladenoff, 2014). The significant increases in N_2_O we found following cultivation suggest that methods of planting that enable establishment with reduced herbicide use could provide benefits of reduced N_2_O emissions. For example, Xue et al. (2017) proposed a method where only small strips of grassland (rather than the entire planting area) are sprayed with herbicide to enable the slot planting of *Miscanthus*. This aims to reduce immediate competition from weeds but also allow continued grassland productivity in the early years of *Miscanthus* establishment. This may provide early season opportunities for grazing prior to the *Miscanthus* shoot emergence in late April/early May.

Fluxes were higher in April 2017 across all the treatments and control compared to the preconversion rates recorded in April 2016, and this early season increase is likely to be related to the slightly higher soil temperature in April 2017. Soil and air temperature also have known links to N_2_O fluxes, often influencing processes that result in a “multiplier effect” on emissions (Butterbach‐Bahl et al., 2013); therefore, the higher temperatures found in the Min Till + Film and No Till treatments (compared to the Pasture) could have had a disproportionate impact on N_2_O emissions. However, despite concerns that the use of a film layer can increase N_2_O emissions (Cuello, Hwang, Gutierrez, Kim, & Kim, 2015; Nishimura, Komada, Takebe, Yonemura, & Kato, 2012), we found that although the film layer did increase soil temperature and NO_3_^‐^ levels and reduce soil moisture, this did not create significantly higher N_2_O emissions compared to the other tillage methods. The film layer also proved to be beneficial for establishment which would therefore contribute to better future yields and hence reductions in yield scaled emissions (Kim, Das, Hwang, & Kim, 2017; Olave et al., 2017).

It was expected that the Min Till treatment would aid establishment and overwinter survivorship compared to the No Till treatment by de‐compacting the soil and allowing easier rhizome/root growth; however, there was no significant difference in the overwinter survivorship between the treatments. Overall survivorship at this upland site (at around 70%) was lower that has been recorded in a nearby lower altitude site (89% at 100 m a.s.l., McCalmont, McNamara, Donnison, Farrar, & Clifton‐Brown, 2017) so it is possible that impacts of tillage were masked by site conditions. However, there are other benefits from no till planting to be considered, such as reduced soil erosion and the retention of soil organic matter (Holland, 2004; Lal et al., 2007). Whilst no till cultivation can sometimes increase N_2_O emissions compared to conventional tillage in wet and poorly aerated soils (Grave et al., 2018; Rochette, 2008), we found no significant difference between the low impact cultivation methods tested.

Although there were increased N_2_O emissions from land use change to *Miscanthus* due to the cultivation of the soil, this is to be expected in the planting of any new crop requiring the killing off of a previous crop and subsequent soil disturbance. There was a clear reduction in emissions over the second growing season suggesting that the higher fluxes (compared to uncultivated pasture) are not likely to last in the long term. The use of the mulch film did not significantly increase N_2_O emissions compared to the other tillage methods tested suggesting that its benefit in extending the possibilities for *Miscanthus* to be grown on agriculturally marginal land does not come at an increased N_2_O cost.
